# Mineral Stabilization of Soil Organic Sulfur at the Continental Scale

**DOI:** 10.1111/gcb.71131

**Published:** 2026-10-04

**Authors:** Zhuojun Zhang, M. Francesca Cotrufo, Benjamin L. Turner, Hai‐Ruo Mao, Chao Liang, Mohsen Shakouri, Mengqiang Zhu

**Affiliations:** ^1^ State Key Laboratory of Lake and Watershed Science for Water Security Nanjing Institute of Geography and Limnology, Chinese Academy of Sciences Nanjing Jiangsu China; ^2^ Department of Ecosystem Science and Management University of Wyoming Laramie Wyoming USA; ^3^ Department of Soil and Crop Science Colorado State University Fort Collins Colorado USA; ^4^ College of Resources and Environment Shandong Agricultural University Tai'an Shandong China; ^5^ Institute of Mountain Hazards and Environment, Chinese Academy of Sciences Chengdu Sichuan China; ^6^ Zhejiang Key Laboratory of Soil Remediation and Quality Improvement, College of Environmental and Resource Sciences Zhejiang A&F University Hangzhou Zhejiang China; ^7^ Institute of Applied Ecology, Chinese Academy of Sciences Shenyang Liaoning China; ^8^ Canadian Light Source Inc. University of Saskatchewan Saskatoon Saskatchewan Canada; ^9^ Department of Geological, Environmental, and Planetary Sciences University of Maryland College Park Maryland USA

**Keywords:** climate change, continental scale, mineral‐associated organic matter, organic sulfur mineralization, sulfur availability, sulfur cycle, sulfur speciation

## Abstract

Declining atmospheric sulfur (S) deposition makes S an emerging limiting macronutrient for plants, yet the stability and dynamics of soil organic S—the largest terrestrial S pool supplying plant‐available sulfate via mineralization—remain unclear. Across North American soils, total organic S is dominated by mineral‐associated organic S (MAOS; 61% ± 26%); this pool is stabilized by mineral protection but saturates at ~600 μg S/g soil. Compared with particulate organic S (POS), which is relatively labile and does not saturate, MAOS is more decomposed, chemically diverse, and climate‐sensitive. Higher temperature accelerates S mineralization in both pools, but their ability to supply available S varies with moisture regime because MAOS stabilizes in wetter soils while POS remains labile. This continental‐scale reassessment of the soil S cycle reveals that secondary minerals play a dual role in soil S cycling by stabilizing organic S and retaining sulfate, providing a mechanistic basis for nutrient management and carbon sequestration in response to climate change in an S‐scarce future.

## Graphical abstract

The conventional whole‐soil view treats soil organic sulfur (S) as a single bulk pool, obscuring the mechanisms governing its transformation, stabilization, and availability. Separating organic S into particulate organic sulfur (POS) and mineral‐associated organic sulfur (MAOS) distinguishes their contrasting sources, microbial transformations, and degrees of mineral protection. In particular, the MAOS–POS framework highlights the central role of soil minerals in stabilizing organic S and retaining sulfate, providing a more mechanistic basis for understanding and predicting soil S persistence and bioavailability under global change.

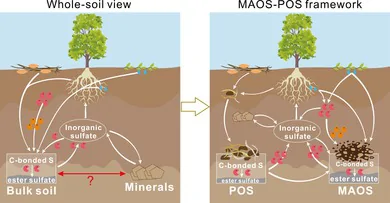

## 1 Introduction

Sulfur (S) is a vital macronutrient essential for all life, yet soils are becoming increasingly deficient in S due to a worldwide decline in atmospheric S deposition (Haneklaus et al. 2008; Hinckley et al. 2020). Soil parent materials are typically low in S, so anthropogenic SO_2_ and ${\mathrm{SO}}_{4}^{2-}$ deposition has been a major external source since the industrial revolution (Haneklaus et al. 2003). However, air quality improvements have reduced S emissions in North America by ~90% (Feinberg et al. 2021; Ritchie and Roser 2021). This decline has led to widespread S limitation across natural and agricultural ecosystems, particularly in arable soils (Hinckley et al. 2020; Messick et al. 2005), prompting growing reliance on supplemental S fertilizers to sustain food production (Gerson and Hinckley 2023; Hinckley et al. 2020).

As atmospheric contributions diminish, microbial mineralization of soil organic S—the largest terrestrial S pool, accounting for over 95% of the total soil S (Eriksen 2009; Kovar and Grant 2011; Niknahad‐Gharmakher et al. 2012)—has become increasingly important for sustaining plant‐available sulfate (Barnard, McKenna, et al. 2025; Churka Blum et al. 2013; Fakhraee et al. 2017; Kovar and Grant 2011; Tolu et al. 2022). Yet organic S compounds are not uniformly accessible: associations with soil minerals can stabilize organic S, limiting microbial access, and constraining mineralization (Eriksen et al. 1998; Solomon et al. 2003; Tanikawa, Hashimoto, et al. 2014; Tanikawa et al. 2022). The balance between microbial mineralization and mineral stabilization has thus emerged as a central, yet poorly understood, control on terrestrial S cycling.

Traditionally, soil organic S has been categorized by biochemical reactivity into labile ester sulfates (C–OSO_3_) and more recalcitrant carbon (C)‐bonded S (C–S), with the former presumed more readily mineralized to meet microbial demand (Churka Blum et al. 2013; Edwards 1998; Eriksen et al. 1998; Singh et al. 2006; Figure 1). However, mineral associations modulate these dynamics by physically protecting both types of compounds from microbial and enzymatic attack (Eriksen et al. 1998; Solomon et al. 2003; Tanikawa, Hashimoto, et al. 2014; Tanikawa et al. 2022; Zhang et al. 2026). Recent developments in soil organic matter research offer a mechanistic framework for soil S: partitioning organic matter into particulate organic matter (POM) and mineral‐associated organic matter (MAOM) has improved predictions of organic C turnover and stability, nitrogen cycling, and responses to climate change (Averill and Waring 2018; Cotrufo et al. 2019; Lavallee et al. 2020; Lugato et al. 2021). Particulate organic matter consists mainly of plant structural residues that decompose on average on decadal timescales and are stabilized by biochemical and microbial constraints, while MAOM consists largely of mineral‐bound soluble or insoluble compounds of either plant or microbial origin with C turnover times ranging from centuries to millennia (Heckman et al. 2021; Lavallee et al. 2020; Liu et al. 2026). This conceptual framework has enhanced our understanding of soil C persistence and nitrogen availability (Averill and Waring 2018; Jilling et al. 2018), yet its relevance to soil organic S remains largely unexplored. Although some studies have demonstrated mineral controls on S dynamics in specific soil types (Eriksen et al. 1998; Solomon et al. 2003; Tanikawa, Hashimoto, et al. 2014; Tanikawa et al. 2022), the quantities, chemical forms, and environmental sensitivities of mineral‐associated organic S (MAOS) versus particulate organic S (POS) remain uncharacterized across ecosystems at broad spatial scales.

**FIGURE 1:**
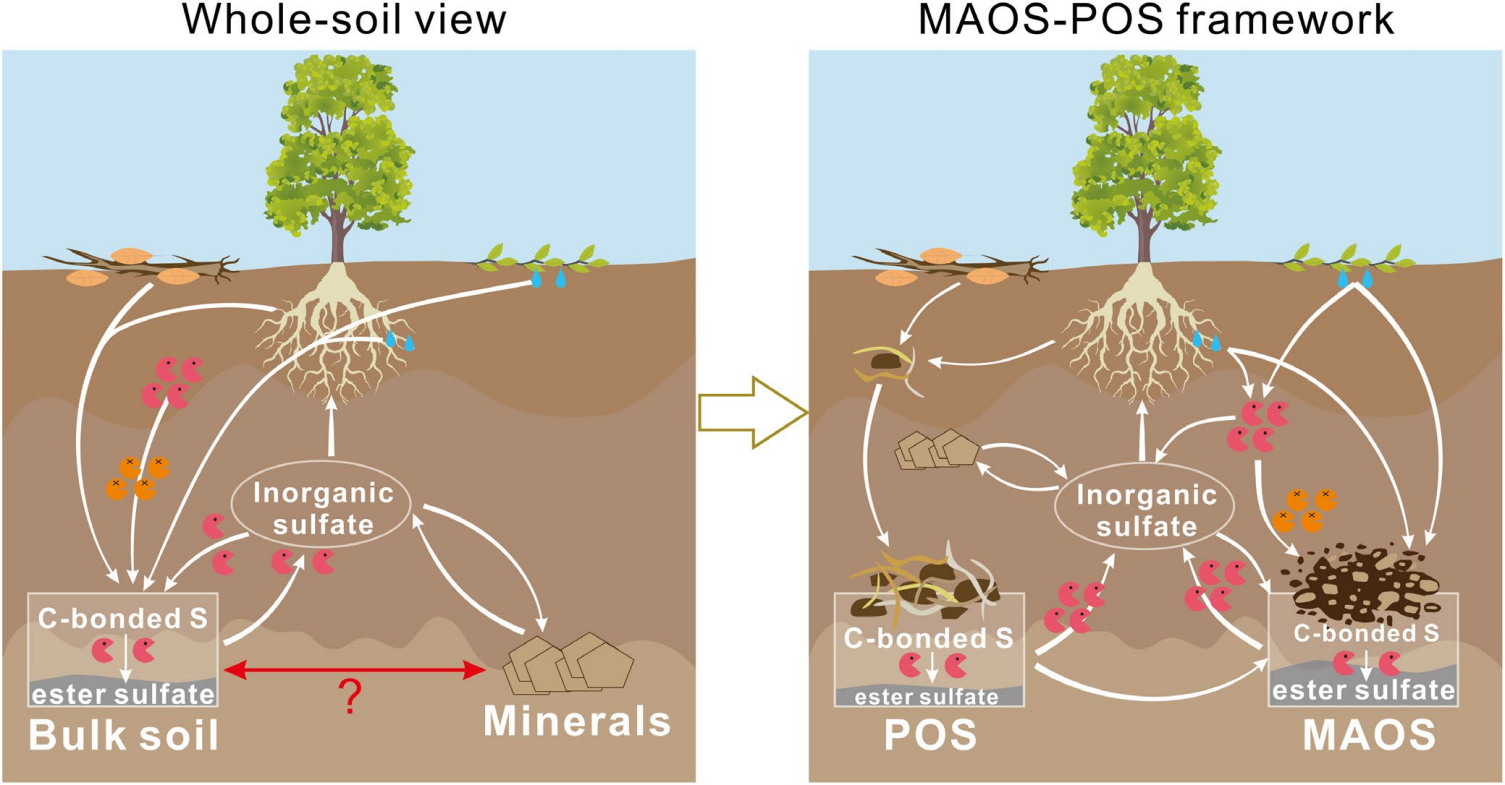
Whole‐soil and two‐pool conceptual frameworks for cycling of soil organic sulfur. Traditional research has treated organic‐S mineralization at the bulk‐soil scale, overlooking the stabilizing role of minerals. The two‐pool framework adds physical resolution by partitioning organic S into particulate organic S (POS), largely derived from fragmented and translocated plant residues, and mineral‐associated organic S (MAOS), formed through sorption of soluble compounds or by microbial transformation and subsequent deposition of microbial residues. Ancillary pathways that influence inorganic sulfate are omitted for clarity.

Here we extend the MAOM–POM framework to S by presenting the first continental‐scale assessment of the physical and chemical nature of soil organic S, using samples from 40 sites across 20 U.S. ecoclimatic domains in the National Ecological Observatory Network (NEON). Consistent with the most recent understanding of organic matter physical fractions (Leuthold et al. 2024), we fractionated soil organic matter by density and size and considered POM as the light (LPOM, < 1.85 g/cm^3^) and coarse heavy fraction (HPOM, > 1.85 g/cm^3^ and > 53 μm), and MAOM as the fine heavy fraction (> 1.85 g/cm^3^ and < 53 μm). LPOM and MAOM were directly characterized, but low S concentrations in HPOM prevented its direct characterization. We measured organic S concentrations in the LPOM (LPOS) and MAOM fractions (MAOS) and the bulk soil (total organic S; TOS). Hereafter, the total particulate organic matter, defined as HPOM + LPOM, is termed POM. Similarly, total particulate organic S contained in combined HPOM and LPOM is denoted as POS, which was estimated as POS = TOS − MAOS. We further characterized S speciation in the MAOM (i.e., MAOS) and LPOM (i.e., LPOS) fractions and the bulk soil using S K‐edge X‐ray absorption near‐edge structure (XANES) spectroscopy. We used average S oxidation state as a proxy for the extent of organic S decomposition and applied path analyses to identify climatic, mineralogical, and other edaphic drivers of S stabilization and decomposition. Our findings provide new insights into the geochemical architecture of soil organic S and its sensitivity to environmental factors, advancing our ability to manage S fertility and availability in a changing climate.

## 2 Materials and Methods

### 2.1 Soil Samples and Analysis

We analyzed A and B horizons from 40 sites using samples in the NEON Megapit archive (National Ecological Observatory Network 2026). The NEON megapit sampling effort was conducted once at each site by NEON staff and the United States Department of Agriculture (USDA) from 2014 to 2018. At each site, a soil pit was excavated to 2 m depth or to bedrock, and horizons were delineated according to the Soil Taxonomy system of classification (Schoeneberger et al. 2012). Most sites contained both A and B horizons. However, RMNP, TEAK, and HEAL were weakly weathered and therefore had A‐C profile development without a clear B horizon, contributing only A‐horizon samples, whereas BONA and PUUM had O‐B‐C profile development without a sampled mineral A horizon and contributed only B‐horizon samples. The study sites span broad geographic, climatic, and ecological gradients across the United States (Figure S1), although soil orders and ecosystem types are unevenly represented. Accordingly, the observed relationships should be interpreted as broad patterns across the sampled environmental gradients rather than as nationally representative estimates. The sites encompass forest, grassland, shrubland, cultivated, and wetland ecosystems, and represent nine soil orders: Alfisols, Andisols, Aridisols, Entisols, Gelisols, Inceptisols, Mollisols, Spodosols, and Ultisols. Within Whittaker biome climate space, the sites cover tundra/boreal forest, temperate seasonal forest, temperate rain forest, temperate grassland/desert, subtropical desert, and woodland/shrubland domains. The number of sites represented by each soil order and ecosystem type is summarized in Figure S1c, and detailed site and soil profile information is provided in Table S1.

Soil pH, particle‐size distribution, bulk density, exchangeable cations, cation exchange capacity, and gypsum data for the sampled horizons were obtained from the NEON Megapit soil physical and chemical properties data product (DP1.00096.001; National Ecological Observatory Network 2026). We determined pedogenic Fe and Al concentrations with a two‐step sequential extraction method. First, 0.1 g soil was extracted with 10 mL of 0.175 M ammonium oxalate (pH 3.2) for poorly crystalline and organic matter‐associated Fe and Al (Schwertmann 1964). The residue was then extracted with 10 mL of citrate‐dithionite solution containing 0.17 g Na_2_S_2_O_4_ and 2 g Na_3_C_6_H_5_O_7_‧2H_2_O for well‐crystallized Fe oxides and Al incorporated into the structure of the Fe oxides (Holmgren 1967). The concentrations of Fe and Al in the ammonium oxalate (Fe_ox_, Al_ox_) and citrate‐dithionite (Fe_cd_, Al_cd_) extracts were measured by inductively coupled plasma‐optical emission spectroscopy (ICP‐OES). Site mean annual temperature (MAT) and mean annual precipitation (MAP) were obtained from WorldClim v2 (Fick and Hijmans 2017), potential evapotranspiration (PET) from Global‐AI_PET v3 (Zomer et al. 2022), and net primary productivity (NPP) from MOD17A3HGF v061 (Running and Zhao 2021). According to the available NEON data, gypsum was absent in A and B horizons at 10 sites (CPER, ONAQ, WREF, DEJU, KONZ, NOGP, BARR, BONA, HEAL, and PUUM), whereas gypsum content was not measured at the remaining 30 sites because soil conditions indicated that gypsum was not present in detectable concentrations.

We used a size/density fractionation scheme to isolate soil organic matter fractions (Soong and Cotrufo 2015). Briefly, a 5 g subsample of oven‐dried bulk soil (60°C) was dispersed with glass beads in 25 mL of 1.85 g/cm^3^ sodium polytungstate solution (SPT). Then, the samples were centrifuged and the light particulate organic matter (LPOM < 1.85 g/cm^3^) was aspirated from the sample and rinsed to remove SPT. The remaining heavy fraction was rinsed thoroughly to remove SPT and then sieved through a 53‐μm screen to separate the heavy sand‐sized organic matter (HPOM > 1.85 g/cm^3^ and > 53 μm) and mineral‐associated organic matter (MAOM > 1.85 g/cm^3^ and < 53 μm). All fractions were oven‐dried at 60°C prior to analysis. The total mass recovery from the fractions was within ±5% of the initial mass.

Total organic C, N, and S concentrations in the LPOM and MAOM fractions and the bulk soil were measured using a Thermo Flash Elemental Analyzer. For each analysis, 5–10 mg of LPOM and 30–60 mg of MAOM or bulk soil were weighed. The method detection limits for C, N, and S were 0.5 g/kg, 0.01 g/kg, and 20 μg/g, respectively. To assess analytical reproducibility, 10% of the samples were analyzed in duplicate, and the resulting analytical precision, expressed as the relative standard deviation, was < 10% for S and < 5% for both C and N. The MAOS concentration in bulk soil was calculated by multiplying the S concentration in MAOM by the mass percentage of MAOM material over total bulk soil. Because HPOM was not directly characterized, total particulate organic S contained in combined HPOM and LPOM is denoted as POS, which was estimated as the difference between TOS and MAOS. The close correlation between LPOS and POS in the topsoil, as indicated by the regression line (Figure S2), suggests that POS is predominantly derived from LPOS. The mineral‐associated organic C (MAOC) and particulate organic C (POC) concentrations were calculated using the same mass‐weighted approach. All S concentrations (TOS, MAOS, and POS) are expressed as μg S/g bulk soil, while C concentrations (MAOC and POC) are expressed as g C/kg bulk soil.

Exchangeable S in soils, considered as available S here, was extracted using a 0.016 M KH_2_PO_4_ solution at a 1:20 ratio. After agitation on a reciprocal shaker for 2 h, the samples were centrifuged and then filtered through 0.45 μm syringe filters. The filtrates were then analyzed for the ${\mathrm{SO}}_{4}^{2-}$ concentration by ion chromatography (Dionex ICS‐90, Thermo Scientific, Massachusetts, USA).

### 2.2 Sulfur K‐edge XANES Spectroscopy

Sulfur K‐edge XANES spectra were collected from bulk soil, LPOM, and MAOM in A and B horizons at the Soft X‐ray Micro‐Characterization Beamline (SXRMB) at the Canadian Light Source, Saskatoon, Canada. All samples were finely ground, homogenized, and mounted as thin, uniform layers. XANES spectra for all samples were recorded in fluorescence yield using a 7‐element SDD detector (RaySpec Inc.) over the energy range of 2442–2542 eV. Measurements were conducted in three segments: 2442–2466 eV with 2 eV steps, 2466–2497 eV with 0.15 eV steps, and 2497–2542 eV with 0.75 eV steps. Self‐absorption was considered minor for these samples because total S concentrations in all samples were < 1.8 mg S/g, far below the ≤ 20 mg S/g range reported for negligible self‐absorption in finely ground materials (Prietzel et al. 2011). The X‐ray energy was calibrated to the K‐edge of elemental S at 2472.7 eV. The XANES spectra were merged and normalized using the software ATHENA. The spectra were further fitted using five Gaussian curves (G1, G2, G3, G4, and G5) that represent the s → p transitions (white line) and two arctangent functions of the transitions of ejected photoelectrons to the continuum (step height or background) following the approach by Wojdyr (2010) using Fityk. The G1 group comprises thiols (R–S–H) and sulfides (R–S–R' and R–S–S–R', etc.), G2 comprises thiophenes (aromatic heterocyclic compounds), G3 comprises sulfoxides (R–SO–R'), G4 comprises sulfonates (R–SO_3_–H), and G5 comprises ester sulfates (R–OSO_3_–H). Peak assignments were based on published S K‐edge XANES reference compounds (Prietzel et al. 2011; Solomon et al. 2003) and are summarized in Table S2. Because Gaussian deconvolution of natural soil spectra cannot uniquely resolve all molecular structures, these assignments should be interpreted as functional group classes rather than compound‐specific identifications.

The relative abundance of each S functional group was calculated from the area under the respective Gaussian peak relative to the total area under all the Gaussian peaks. The areas were corrected for energy‐dependent absorption cross section using the generic calibration curve (Manceau and Nagy 2012):

(1) y=0.36841x−909.97

where *x* is the white‐line peak energy and *y* is the scaling factor normalized to *y* = 1 at the energy of elemental S (E = 2472.70 eV). It has been reported that the error for the organic S fraction with the Gaussian curve fit is 4%–8% for reduced and < 5% for oxidized S species (Huffman et al. 1991; Manceau and Nagy 2012).

The electronic oxidation state (EOS) of each S species was calculated using the equation (Zeng et al. 2013):

(2) EOS=0.6179x−1529

where *x* is the white‐line peak energy. EOS values were used to describe the apparent oxidation state of the fitted functional group classes, not to assign individual molecular species. In this study, EOS values were reported as calculated for the low‐valent S species (EOS ≤ +4) and EOS values were rounded to integer values for the high‐valent S species (EOS > +4). The average S oxidation state (ASOX) was calculated using the following equation:

(3) $$\begin{array}{c} \text{ASOX}={\mathrm{EOS}}_{\text{G1}}\times F_{\text{G1}}+{\mathrm{EOS}}_{\text{G2}}\times F_{\text{G2}}+{\mathrm{EOS}}_{\text{G3}}\times F_{\text{G3}}+{\mathrm{EOS}}_{\text{G4}}\\ \;\times F_{\text{G4}}+{\mathrm{EOS}}_{\text{G5}}\times F_{\text{G5}} \end{array}$$

where F represents the relative proportion of each Gaussian component (G1–G5).

In this study, XANES‐derived S speciation was expressed as relative fractions rather than converted to species‐specific concentrations because concentration‐weighted values would combine variation in total organic S pool size with variation in relative S speciation. We therefore evaluated organic S pool size using MAOS and POS concentrations, and S chemical composition using species fractions and average S oxidation state.

### 2.3 Statistical Analyses

Structural equation modeling (SEM) was used as a path‐analysis framework to evaluate the direct and indirect effects of climatic, edaphic, mineralogical, and organic matter variables on organic S pools, S oxidation states, and sulfate availability. The target response variables included the concentrations of MAOS and POS, the proportion of MAOS in total organic S (f_MAOS_), exchangeable S concentration and percentage of total soil S, as well as average S oxidation states in bulk soil, LPOM, and MAOM. For models of MAOS, POS, f_MAOS_, and average S oxidation states, candidate predictor variables included MAT, effective soil moisture defined as mean annual precipitation minus potential evapotranspiration (MAP‐PET), ammonium oxalate‐extractable Fe and Al concentration, silt and clay content, pH, MAOC concentration, POC concentration, proportion of MAOC in total organic C (f_MAOC_), and the C/N ratios of bulk soil, LPOM, and MAOM. For models of exchangeable S concentration and percentage, candidate predictors included MAT, MAP‐PET, FeAl_ox_ concentration, silt and clay content, pH, MAOS concentration, POS concentration, and average S oxidation states of LPOM and MAOM. Unlike regression or analysis of variance, SEM offers the ability to separate multiple pathways of influence and view them as a system. Thus, SEM is useful for investigating the complex networks of relationships found in natural ecosystems (Grace 2006; Shipley 2016). Each path coefficient (λ) represents the standardized effect of a predictor on a target variable and is analogous to a standardized regression coefficient. The total effects were obtained by multiplying the λ values along the related effect paths and summing them. The *R* ^2^ values represented the proportion of the variance in each response variable explained by the model. Multiple goodness‐of‐fit tests were applied, including the Chi‐square/degree of freedom (χ^2^/df) test (*p* > 0.05), the comparative fit index (CFI > 0.9), and the root mean square error of approximation (RMSEA < 0.08). The SEM analyses were conducted using AMOS 22.0 (Amos Development Co.).

All the correlation analyses in this study were conducted using Origin 2018. Differences in S concentrations and C/S and C/N ratios among bulk soil, POM, and MAOM fractions and across horizons or ecosystem types were tested using one‐way analysis of variance (ANOVA) followed by Tukey's honestly significant difference test for post hoc comparisons. All correlations among variables reported in this study were fitted using ordinary least squares linear regression, except for the relationships of TOS with MAOS and POS concentrations, which were fitted using asymptotic and ExpDec models, respectively.

## 3 Results and Discussion

### 3.1 Distributions of MAOS and POS

We find that MAOS constitutes a substantial fraction of total organic S across North American soils, though its contribution varies considerably across sites (Figure 2a,b). In the surface (A) horizon, mean (±SD) concentrations of TOS, MAOS, and POS were 312 ± 294, 155 ± 124, and 157 ± 259 μg/g soil, respectively, declining to 187 ± 231, 88 ± 84, and 99 ± 199 μg/g soil in the B horizon (Figure 2a). The fraction of MAOS relative to TOS (f_MAOS_) averaged 57% ± 21% in the A horizon soil and 64% ± 28% in the B horizon (subsurface), without a significant difference between the two horizons (Figure 2b). On average, mineral‐associated S accounted for 61% ± 26% of total organic S across all samples, underscoring the widespread role of mineral stabilization in shaping soil organic S pools and identifying MAOM as, on average, a larger S reservoir than POM. Surprisingly, the concentrations of TOS, MAOS, and POS as well as f_MAOS_ did not differ significantly among ecosystem types (Figure S3a–d), suggesting common underlying mechanisms governing S partitioning across ecosystems although the unequal numbers of sites per ecosystem type limit the power of this comparison.

**FIGURE 2:**
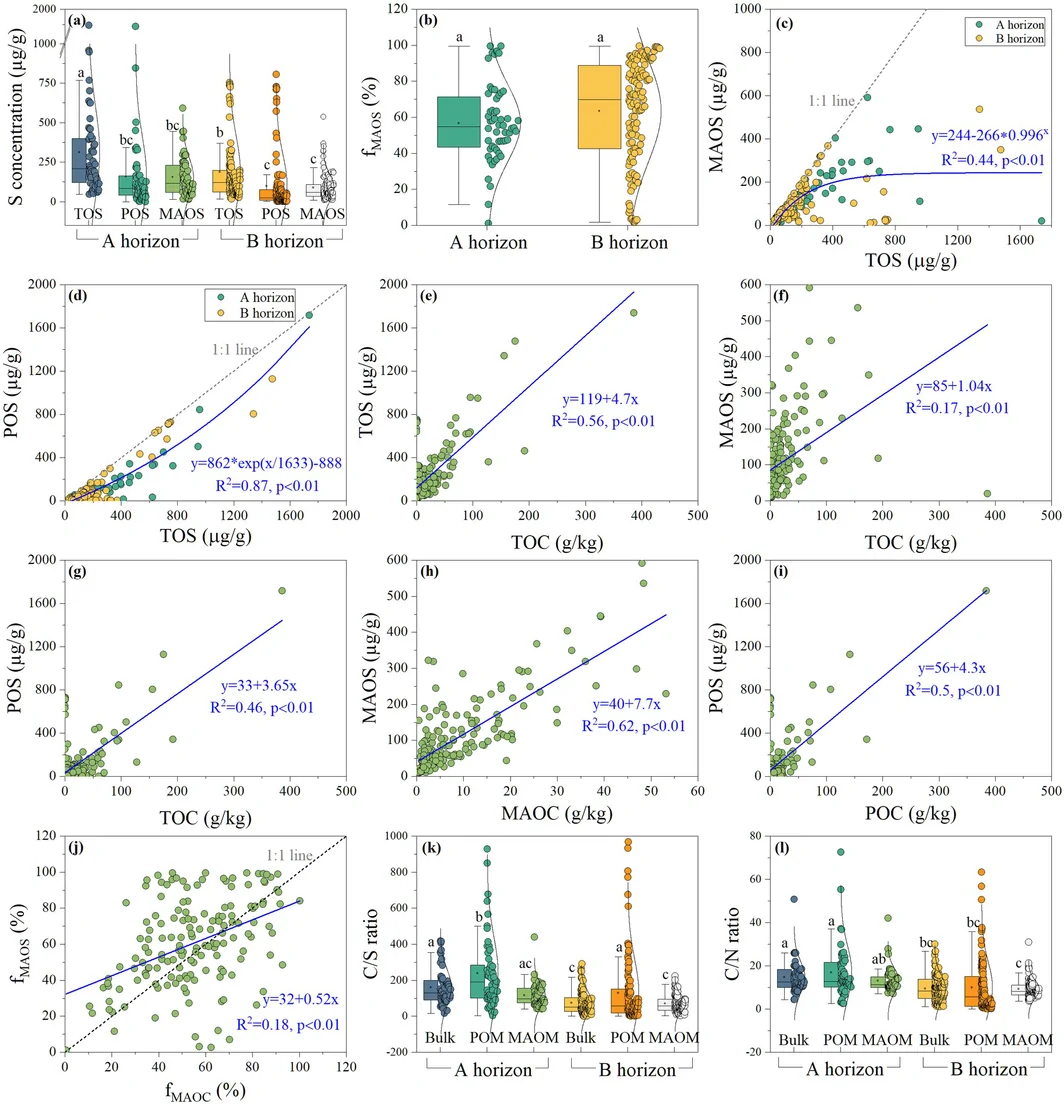
Distributions of mineral‐associated organic S (MAOS) and particulate organic S (POS). (a) Distribution of organic S in bulk soil (TOS), POS and MAOS fractions in the A and B horizons. (b) Fractions of MAOS (f_MAOS_) in the A and B horizons. (c, d) Correlations of MAOS and POS concentrations with TOS in the A and B horizons. (e, f, g) Correlations of TOS, MAOS, and POS with TOC. (h, i, j) Correlations between MAOS and mineral‐associated organic C (MAOC), POS and particulate organic C (POC), and f_MAOS_ and fractions of MAOC (f_MAOC_). (k, l) The C/S and C/N ratios in bulk soil, POM and MAOM fractions in the A and B horizons. POM is the sum of light POM and heavy POM. All S concentrations are expressed as μg S/g bulk soil, and all C concentrations as g C/kg bulk soil.

Despite the widespread contribution of MAOS to soil organic S, the capacity of soils to stabilize organic S through mineral associations appears finite. MAOS concentrations exhibit a saturation response with increasing TOS: at low TOS levels, sulfur was primarily stored as MAOS (Figure 2c), but beyond ~600 μg S/g soil, MAOS reached an apparent upper limit, and further increases in TOS occurred exclusively via POS accumulation (Figure 2d). This pattern closely parallels MAOC saturation (Figure S4; Cotrufo and Lavallee 2022; Cotrufo et al. 2019), as expected given the consistently narrow C/S ratios in MAOM (Figure 2k). The MAOS saturation limit of ~600 μg S/g soil corresponds to a theoretical MAOC limit of ~60 g C/kg soil (mean C/S ratio is 100:1, Figure 2k). This calculated threshold aligns quantitatively with MAOC saturation levels (40–75 g C/kg soil) reported across European grasslands and forests (Cotrufo et al. 2019). Assuming a typical theoretical C loading capacity of ~1 mg C/m^2^ of mineral surface (Mayer 1994), this saturation limit implies an effective specific surface area of ~60 m^2^/g soil, which is characteristic of fine‐textured soils rich in clay and reactive metal oxides (Kaiser and Guggenberger 2003; Wagai et al. 2009). Thus, the MAOS limit appears to be fundamentally constrained by the availability of mineral surfaces. However, confirming the saturation threshold requires further empirical work, such as isotopic tracing and controlled S enrichment studies, to assess incorporation rates and stabilization capacity.

Our findings suggest that S availability and mineral interactions may jointly stabilize organic C in soils, underscoring the potential biogeochemical coupling of organic S to organic C. A comparison of C/S ratios between MAOM and POM suggests that S availability constrains MAOC formation. Similar to the C/N ratio, MAOM consistently exhibited lower and less variable C/S ratios than POM (Figure 2k,l), reflecting more advanced microbial processing, a higher S demand for MAOM formation (Kirkby et al. 2013), and/or a stronger affinity of S‐rich organic molecules for soil mineral surfaces (Spohn 2024). Soils with higher f_MAOC_ tended to show lower C/S ratios in MAOM (Figure S6), suggesting lower S use efficiency with increasing stabilization of soil organic matter. These patterns imply that, like nitrogen and phosphorus (Favaro et al. 2025; Lieberman et al. 2025; Spohn 2020), sulfur might limit long‐term organic C stabilization, an effect likely to intensify as atmospheric S inputs continue to decline. The close relationship between organic C and S is further supported by strong correlations between MAOC and MAOS, and between POC and POS (Figures 2h–i and 3a–b). Although TOS, MAOS, and POS increased with bulk total organic C, MAOS and POS were more strongly related to their corresponding C pools than to bulk total organic C (Figure 2e–i), indicating that organic S accumulation reflects not only total organic matter abundance but also its partitioning between mineral‐associated and particulate pools.

**FIGURE 3:**
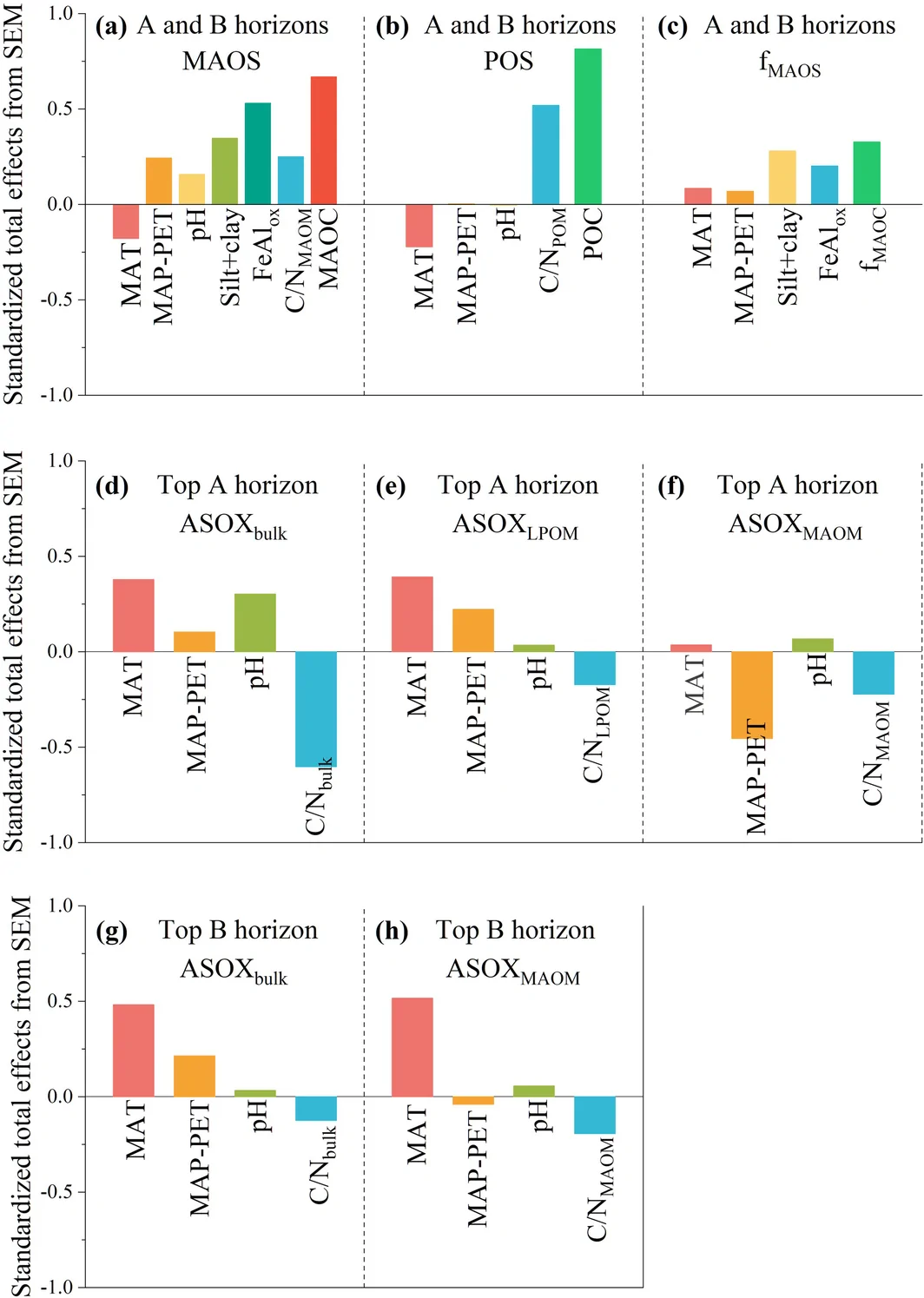
Standardized total effects (direct plus indirect effects) derived from the structural equation modeling (SEM). These effects show the controls on: (a–c) mineral‐associated organic S (MAOS) concentrations, particulate organic S (POS) concentrations, and proportion of MAOS in total organic S (f_MAOS_) in the A and B horizons; (d–f) average S oxidation states (ASOX) in bulk soil, LPOM and MAOM in the top A horizons; and (g, h) ASOX in bulk soil and MAOM in the top B horizons. Predictor variables include mean annual temperature (MAT), effective soil moisture (mean annual precipitation minus potential evapotranspiration, that is, MAP‐PET), mineral‐associated organic C (MAOC) concentration, particulate organic C (POC) concentration, proportion of MAOC in total organic C (f_MAOC_), ammonium oxalate‐extractable Fe and Al (FeAl_ox_) concentration, silt and clay (silt+clay) content, pH, C/N ratio of bulk soil (C/N_bulk_), C/N ratio of light particulate organic matter (C/N_LPOM_), C/N ratio of mineral‐associated organic matter (C/N_MAOM_). The amount of LPOM material in B horizons was not sufficient for characterization. The full SEM path diagrams are shown in Figure S14.

MAOS and POS concentrations were elevated in soils with higher C/N ratios of MAOM and POM and in cooler climates (Figure 3a,b), suggesting potential S enrichment in less decomposed organic matter. This likely reflects the relative stability of C‐bonded S in fresher material compared to ester‐bound S in more processed compounds. Mineralogical controls were especially important for MAOS: both silt/clay content and oxalate‐extractable Fe and Al were positively associated with MAOS concentrations (Figure 3a), although the strength of these relationships varied between A and B horizons (Figure S7), highlighting the role of secondary minerals in organic S accumulation and stabilization. Higher effective soil moisture may further enhance MAOS accrual, likely by stimulating mineral weathering and plant productivity, while lower pH may suppress microbial processing and necromass stabilization. MAOS and POS concentrations were largely independent of depth, although greater horizon thickness increased horizon‐scale MAOS and TOS stocks (Figure S8). Among all variables, the fraction of MAOC (f_MAOC_) emerged as the strongest predictor of the proportion of MAOS in total organic S (f_MAOS_; Figure 3c), consistent with the observed strong correlation from the linear regression analysis (Figure 2j).

### 3.2 Oxidation States and Decomposition Degree of MAOS and POS

The chemical composition of organic S varied widely across the continent and differed significantly between MAOS and LPOS, though not among ecosystems (Figure 4A; Figure S3j–r). Light POS in the A horizon was dominated by C‐bonded S (89% ± 4%) with little ester sulfate (11% ± 4%), whereas MAOS exhibited lower C‐bonded S (73% ± 13%) and higher ester sulfate (27% ± 13%) with greater variability (as shown by higher standard deviations) than POS (Figure 4A), reflecting a more oxidized and decomposed state of MAOS. This greater variability suggests that MAOS is more chemically sensitive than LPOS to environmental conditions. This finding is consistent with reports that organic S is more oxidized in clay fractions than in bulk soils (Barnard, Dalal, et al. 2025; Prietzel et al. 2007). Despite being more decomposed on average, MAOS is expected to be more stable due to mineral protection, particularly for ester sulfate through strong adsorption of its ${\mathrm{SO}}_{4}^{2-}$ group onto mineral surfaces. In addition to direct adsorption of the ${\mathrm{SO}}_{4}^{2-}$ group on positively charged mineral surfaces such as metal oxides, cation bridging may be involved in its adsorption on negatively charged phyllosilicates. This plausible stabilization mechanism is consistent with positive correlations of ester sulfate content in the MAOM fraction with exchangeable Ca^2+^ and Mg^2+^ and soil cation exchange capacity (CEC; Figure S15). All these interactions can slow mineralization of the organic S (Prietzel et al. 2007; Tanikawa, Noguchi, et al. 2014; Yang et al. 2016).

**FIGURE 4:**
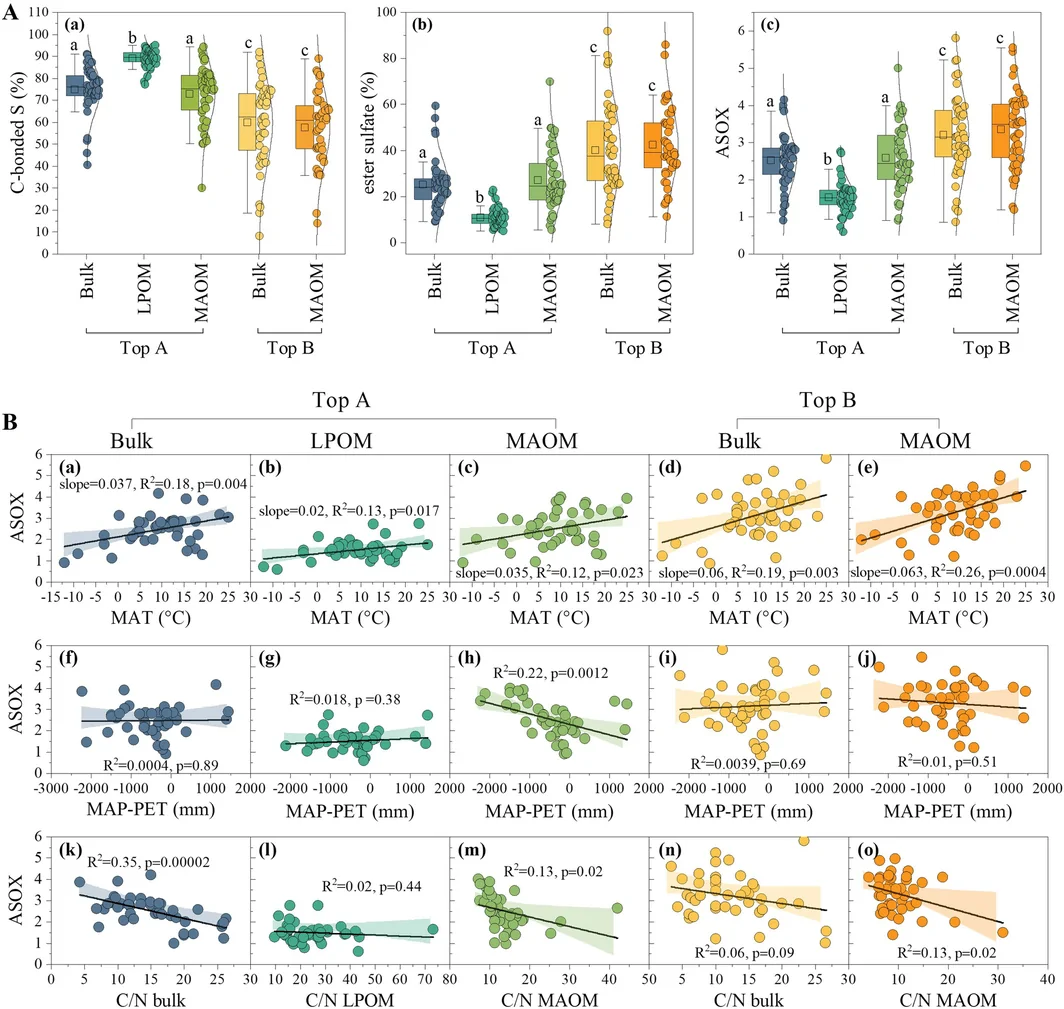
Chemical composition and oxidation states of MAOS and LPOS. (A) The chemical composition and average S oxidation states (ASOX) in bulk soil, light particulate organic matter (LPOM) and mineral‐associated organic matter (MAOM) in the top A and B horizons, and (B) their correlations with mean annual temperature (MAT), effective soil moisture (mean annual precipitation minus potential evapotranspiration, i.e., MAP‐PET), and the C/N ratio.

The contrasting compositions of MAOS and LPOS reflect differences in the origin and transformation of MAOM and LPOM. The high C‐bonded S content in LPOM aligns with its predominantly plant‐derived, less‐decomposed nature, as plants contribute C‐bonded S primarily through amino acids and proteins (Turner et al. 2016). Microbial biomass also contributes reduced C‐bonded S, largely as amino acids and proteins, although fungal residues may contain a relatively large proportion of ester‐bonded S, likely as choline sulfate (Heinze et al. 2021). Within LPOM, oxidation of this C‐bonded S produces ester sulfate, which can then hydrolyze rapidly to release inorganic sulfate because it lacks mineral protection. Thus, oxidation and subsequent rapid hydrolysis of organic S further enrich C‐bonded S in LPOM relative to ester sulfate. In contrast, although C‐bonded S remains the dominant form in MAOM, MAOM contains a higher and more variable proportion of ester sulfate than LPOM, which likely reflects both greater microbial processing and stronger mineral stabilization. As plant‐ and microbial‐derived organic matter is decomposed and transformed, C‐bonded S may be progressively converted into more oxidized S forms including ester sulfate. Ester sulfate may also accumulate in MAOM through selective sorption of ester‐sulfate‐containing dissolved organic matter onto mineral surfaces. Once associated with MAOM, ester sulfate may be protected against further hydrolysis or mineralization through preferential adsorption to mineral surfaces via its ${\mathrm{SO}}_{4}^{2-}$ group. The higher ester sulfate proportion in B horizon soils (Figure 4Ab) further supports the role of mineral surfaces, particularly of secondary minerals abundant in deeper layers, in concentrating and stabilizing ester sulfate.

Environmental conditions, especially temperature and effective soil moisture, appear to further shape the chemical state and stability of organic S pools. Path analyses identified MAT, MAP‐PET, and soil pH (itself influenced by MAP‐PET; Figure S9b) as potential key drivers. MAT positively correlated with the oxidation states of both MAOS and LPOS, with stronger effects in the B horizon than the A horizon (Figures 3d–h and 4Ba–e), consistent with evidence that warming accelerates organic matter decomposition more in subsoils (Jia et al. 2019; Ofiti et al. 2021). Species‐level analyses further supported this pattern: reduced S fractions generally declined with increasing MAT, whereas ester sulfate increased (Figure S12). In contrast, MAP‐PET had divergent effects on the two S pools (Figures 3d–h and 4Bf–j). LPOS oxidation increased slightly with MAP‐PET, likely due to enhanced decomposition in wetter but oxygen‐rich macropores hosting LPOM, though this effect might be tempered by lower pH at high moisture. MAOS oxidation, however, declined markedly with increasing MAP‐PET, especially in the A horizon. This pattern was also evident at the species level, where increasing MAP‐PET was associated with greater reduced S fractions and lower ester sulfate in MAOM of the A horizon (Figure S13k–o). While these NEON bulk soils did not show significant correlations between average S oxidation state and MAP‐PET (Figure 4Bf), a similar decline in average S oxidation state was observed in bulk soils across a well‐constrained rainfall gradient in the Hawaiian Islands (Zhu et al. 2026). Increased leaching under wetter conditions may transport less‐decomposed, plant‐derived compounds into mineral soils, enriching MAOM in reduced organic S (Kalbitz and Kaiser 2008; Yu et al. 2022). Consistently, Liu et al. (2026) showed across North American soils that the soluble component of MAOC is enriched in plant‐associated aromatic compounds and contributes more strongly to MAOC under wetter, more acidic, Fe/Al‐rich conditions. Wetter conditions may also enhance anoxia and acidification in micropores hosting MAOM, which limit microbial oxidation and preserve reduced S forms (Keiluweit et al. 2017; Zhu et al. 2026). Compared to the A horizon, the B horizon showed weaker correlations with MAP‐PET, likely because organic matter inputs via leaching are lower (Camino‐Serrano et al. 2014; Michalzik et al. 2001), while lower organic substrate availability (Fontaine et al. 2007), reduced microbial activity (Min et al. 2021), and a weaker coupling between surface climatic water balance and local redox dynamics (Keiluweit et al. 2018) collectively desensitize microbial oxidation to variations in moisture. Overall, these results demonstrate how climate‐driven variations in temperature and moisture modulate organic S oxidation and stabilization, with implications for the persistence of organic S and release of plant‐available sulfate in soils.

### 3.3 Sulfate Availability in Soils

Sulfur availability in soils, typically measured as exchangeable S, averaged 12 ± 13 μg/g soil in the A horizon and 41 ± 80 μg/g soil in the B horizon (Figure 5a). The proportion of exchangeable S relative to total S (f_exchangeable S_) was also higher in the B horizon (20% ± 21%) than in the A horizon (5% ± 6%; Figure 5b). Both the concentration and proportion of exchangeable S were consistently greater in forest ecosystems compared to grasslands (Figure S3e,f).

**FIGURE 5:**
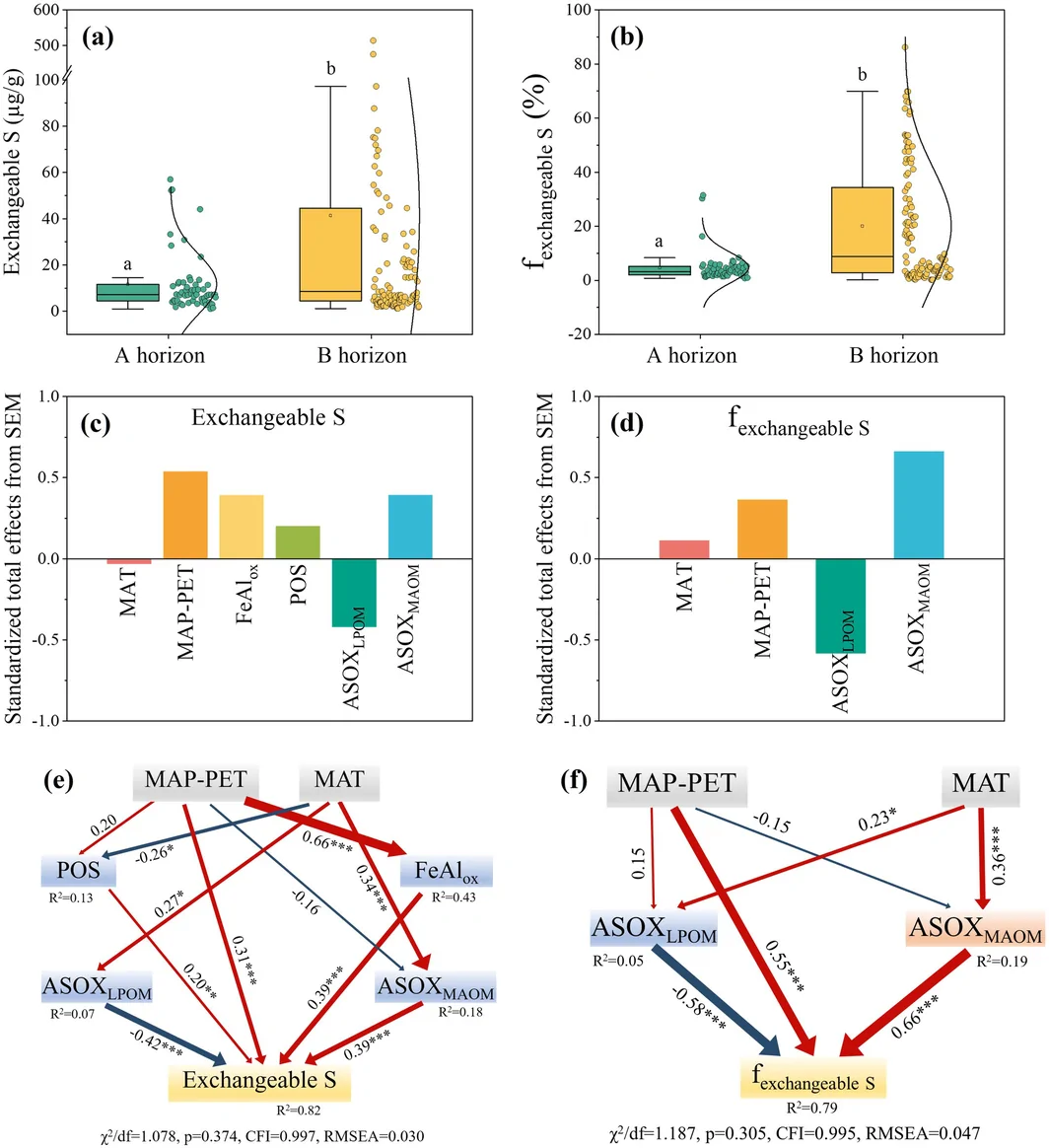
Sulfate availability and effects. (a, b) Concentration and proportion of exchangeable S in the A and B horizons. (c, d) Standardized total effects (direct plus indirect effects) derived from the structural equation modeling (SEM) identifying the controls on exchangeable S concentration and percentage in the A and B horizons. (e, f) SEM path diagrams for exchangeable S concentration and percentage. Numbers adjacent to arrows are standardized path coefficients, analogous to standardized regression coefficients. Red and blue arrows indicate positive and negative relationships, respectively. Arrow width is proportional to the strength of the relationship. The proportion of variance explained (*R* ^2^) is shown next to each response variable. Goodness‐of‐fit statistics are shown below each model, including Chi‐square/degree of freedom (χ^2^/df) test, the comparative fit index (CFI), and the root mean square error of approximation (RMSEA). *p < 0.05, **p < 0.01, ***p < 0.001. ASOX_LPOM_, average S oxidation states of light particulate organic matter; ASOX_MAOM_, average S oxidation states of mineral‐associated organic matter; exchangeable S, exchangeable S concentration; f _exchangeable S_, exchangeable S percentage; FeAl_ox_, ammonium oxalate‐extractable Fe and Al concentration; MAP‐PET, effective soil moisture; MAT, mean annual temperature; POS, particulate organic S concentration.

Exchangeable S in soils is influenced by climatic conditions and soil mineralogy, with positive correlations with MAP‐PET and oxalate‐extractable Fe and Al (Figure 5c). The strong association with Fe and Al underscores the role of sulfate adsorption to metal oxides, which explains the higher exchangeable S levels in the B horizon and in forest soils, where these oxides are more abundant (Figure 5a; Figure S3e). MAP‐PET likely enhances exchangeable S by promoting Fe/Al oxide formation and increasing wet atmospheric sulfate deposition, though dry deposition, which may also contribute (Luo et al. 2016), was not considered in this study.

Beyond environmental drivers, the size and oxidation state of organic S pools may play key roles in S availability. Exchangeable S was positively correlated with POS concentration and the oxidation state of MAOS, but negatively with the oxidation state of LPOS (Figure 5c). The link to POS may reflect the high lability and rapid turnover of POS, which readily releases sulfate into the exchangeable pool. The negative correlation with the oxidation state of LPOS likely reflects the loss of ester sulfate through hydrolysis, which both lowers its oxidation state and contributes sulfate to the exchangeable pool. That is, oxidative decomposition of LPOS is an important source of exchangeable sulfate. In contrast, the positive correlation with the oxidation state of MAOS suggests that ester sulfate‐rich MAOS, although stabilized by mineral associations, can gradually release sulfate over time through enzymatic mineralization following MAOM destabilization. The same factors and mechanisms influencing exchangeable S concentrations also apply to its proportion relative to total S, except that POS concentration and Fe/Al oxides showed much weaker effects (Figure 5d). Overall, oxidation states of organic S appeared to be an important correlate of exchangeable sulfate, alongside organic S pool sizes. This finding suggests that the degree of organic matter decomposition may provide a useful indicator of S availability, although declining atmospheric deposition of sulfate may complicate this relationship.

### 3.4 Role of Minerals in Sustaining Sulfur Fertility in Soils

Our findings indicate that minerals in the silt‐ and clay‐sized fractions exert dual control on soil S availability. On one hand, mineral associations concentrate organic S in MAOM and protect it from microbial decomposition, particularly organic sulfate, presumably slowing down the release of inorganic sulfate for biological uptake. On the other hand, the same minerals adsorb inorganic sulfate with moderate binding strength, reducing leaching while maintaining its plant availability (Eriksen and Askegaard 2000). Elevated exchangeable sulfate in clay‐rich soils therefore indicates that, despite protection, MAOS continues to mineralize slowly as MAOM is destabilized (Bölscher et al. 2025; Jilling et al. 2025); the released sulfate is efficiently retained on mineral surfaces and accumulates over time. Inorganic sulfate inputs from mineralization of POS and atmospheric deposition also enhance the adsorbed sulfate pool in soils. Consequently, sulfate deficiency is common in sandy soils where low adsorption capacity facilitates organic S mineralization and subsequent sulfate leaching, but rare in fine‐textured soils (Edwards 1998). Thus, minerals function as sustained slow‐release agents of plant‐available sulfate in soils from the perspective of both organic and inorganic S pools.

### 3.5 Implications for Sulfur Nutrient Management and Limitations

Our results predict that projected warming will accelerate S mineralization from organic S pools, but that shifting moisture regimes will drive their divergence in supplying available S. Ignoring these differences between the two pools in response to climate change risks yielding inaccurate predictions and poor management strategies. Our findings suggest that sulfate release from MAOS is greater in regions projected to become warmer and drier (Dai 2013), heightening the risk of S leaching into aquatic ecosystems, which can trigger severe environmental cascades such as mercury methylation, eutrophication, and sulfide toxicity to native flora and fauna (Lamers et al. 2002; Regnell and Watras 2019; Smolders and Roelofs 1993; Wang and Chapman 1999). In contrast, wetter ecosystems (O'Gorman 2015) may sequester more S in MAOS, while seasonal wet–dry cycles or climate‐driven hydrological shifts may substantially affect sulfate availability in these systems (Liu et al. 2021).

While derived from natural ecosystems, our findings offer a framework for fertility management in agricultural systems that leverages both POS and MAOS pools through practices tailored to soil texture. In coarse‐textured soils, organic inputs (e.g., crop residues, cover crops, and compost) build a labile POS pool for timely sulfate release (Barnard, McKenna, et al. 2025; Cardelli et al. 2008; Hansen et al. 2021; Piutti et al. 2015), while Fe‐ and Al‐rich amendments or biochar expand MAOS, retain sulfate and limit its leaching (Ding et al. 2016; Lei et al. 2024; Penn et al. 2011). Conversely, fine‐textured (clay‐rich) soils require moderate aeration and controlled MAOS turnover (via brief drainage or shallow fertilizer banding) to release sulfate without depleting long‐term S reserves (Tariq et al. 2018; Zheng et al. 2024). Notably, any S management strategy must be balanced with C sequestration goals. Because the mineral surfaces that stabilize MAOS also protect organic C, aggressively mobilizing sulfate can oxidize stored C, whereas maximizing soil organic C storage (e.g., via clay addition or no‐till) can immobilize S and induce deficiencies (Janzen 2006; Wright and Hons 2005). Thus, an optimal strategy is to maintain a predominantly MAOC–MAOS matrix while creating localized, temporal hotspots of S mineralization (through banded fertilizer, rhizosphere redox shifts, or strip tillage; Battisti et al. 2022; Bölscher et al. 2025; Brainard et al. 2013; Tauchnitz et al. 2018) so that roots capture released sulfate and prevent S deficiencies from undermining C sequestration efforts.

Several limitations should be considered when interpreting the implications of these continental‐scale findings. The climatic, mineralogical, and organic matter variables explained only part of the variation in organic S partitioning and oxidation state (Figure S14), indicating that important controls remain unresolved. These may include site‐specific S deposition history, parent material sulfate or gypsum, microbial S cycling capacity, vegetation S chemistry, hydrological and redox history, and exchangeable cation composition (Karimian et al. 2018; Kertesz and Mirleau 2004; Luo et al. 2016; Ng et al. 2017; Tanikawa et al. 2022; Turner et al. 2016; Zhang et al. 2024; Zhang et al. 2021). Although exchangeable S concentrations were generally low, gypsum content was unavailable for many sites. Unmeasured gypsum or other inorganic sulfate minerals may therefore have influenced TOS and f_MAOS_ estimates in some dryland soils, although they are unlikely to explain the overall continental‐scale patterns. Moreover, the dataset integrates soils with diverse parent materials, soil orders, textures, vegetation types, and land‐use histories, which may weaken cross‐site correlations and mask stronger relationships within specific soil groups or regions. Limited sample sizes and narrow climatic ranges within many soil orders further constrain the detection of within‐group trends (Figure S16A). More focused analyses within individual soil orders or climate regions are needed to resolve these context‐specific controls. Finally, because the dataset is largely restricted to the continental United States and primarily represents temperate and semi‐arid ecosystems, with limited tropical and subtropical coverage, the climate‐ and moisture‐related relationships observed here should be interpreted with caution in underrepresented systems.

Despite these limitations, our study establishes a continental‐scale framework for soil organic S cycling by resolving its partitioning between particulate and mineral‐associated pools. By linking these pools and their speciation to organic C, mineral properties, and climate, this framework advances understanding of S cycling and availability in soils and provides a basis for improving biogeochemical models and S management.

## Author Contributions

**Zhuojun Zhang:** writing – original draft, writing – review and editing, data curation, formal analysis, conceptualization, methodology, software, validation, visualization, investigation. **M. Francesca Cotrufo:** writing – review and editing, methodology. **Benjamin L. Turner:** writing – review and editing, formal analysis. **Hai‐Ruo Mao:** writing – review and editing, methodology, data curation. **Chao Liang:** writing – review and editing. **Mohsen Shakouri:** writing – review and editing, methodology. **Mengqiang Zhu:** conceptualization, methodology, data curation, validation, formal analysis, supervision, resources, project administration, funding acquisition, writing – original draft, writing – review and editing.

## Funding

This work was supported by the U.S. National Science Foundation, DEB‐2027284, EAR‐1752903.

## Conflicts of Interest

The authors declare no conflicts of interest.

## Supporting information


**Data S1:** Sulfur XANES spectra data.

**Figure S1:** Spatial distribution and Whittaker biome classification of the study sites. (a) Geographic distribution of sites, with symbols indicating soil order and colors indicating vegetation type. Map lines delineate study areas and do not necessarily depict accepted national boundaries. (b) Whittaker biome distributions of all sites (Whittaker, 1970). (c) Number of sites represented by each soil order and ecosystem type.
**Figure S2:** Correlation between LPOS and POS in the topsoil.
**Figure S3:** A comparative analysis of key organic S and soil characteristics across different ecosystems. (a–d) Total organic S (TOS), particulate organic S (POS), mineral‐associated organic S (MAOS), and the proportion of MAOS in TOS (f_MAOS_); (e, f) exchangeable S concentration and its proportion of total S; (g–i) C/S ratios of bulk soil, particulate organic matter (POM), and mineral‐associated organic matter (MAOM); (j‐l) C‐bonded S, ester sulfate, and average S oxidation state (ASOX) in bulk soil; (m–o) C‐bonded S, ester sulfate, and ASOX in light particulate organic matter (LPOM); (p–r) C‐bonded S, ester sulfate, and ASOX in MAOM; and (s–x) ammonium oxalate‐extractable Fe and Al, dithionite‐extractable Fe and Al, pH, soil organic C (SOC), C/N ratio, and silt + clay content, respectively. Different lowercase letters indicate significant differences among ecosystem types (one‐way ANOVA followed by Tukey's HSD, *p* < 0.05).
**Figure S4:** Correlations between TOC (g/kg soil) and concentrations of MAOC and POC (g/kg soil) in the A and B horizons.
**Figure S5:** Correlations between C/S and C/N ratios, as well as N/S and C/N ratios, in bulk soil, POM, and MAOM fractions.
**Figure S6:** Correlation between f_MAOC_ and C/S_MAOM_.
**Figure S7:** Relationships of MAOS concentration with (a) silt and clay (silt+clay) content and (b) ammonium oxalate‐extractable Fe and Al (FeAl_ox_) concentration.
**Figure S8:** Relationships between organic S pools and horizon thickness/depth. For A, B, and A + B horizons, MAOS, POS, and TOS stocks were calculated as organic S concentration × bulk density × horizon thickness.
**Figure S9:** (a) Correlation between NPP and MAT, and (b) correlation between soil pH and MAP‐PET.
**Figure S10:** The chemical composition of organic S groups in bulk soil, LPOM and MAOM in the top A and B horizons.
**Figure S11:** Correlations of the proportions of C‐bonded S and ester sulfate with MAT and MAP‐PET in bulk soil, LPOM, and MAOM across the top A and B horizons.
**Figure S12:** Correlations of the proportions of the five Gaussian components (G1—G5) with MAT in bulk soil, LPOM, and MAOM across the top A and B horizons. G1 represents reduced organic S, G2 thiophene‐like S, G3 sulfoxide, G4 sulfonate, and G5 ester sulfate.
**Figure S13:** Correlations of the proportions of the five Gaussian components (G1—G5) with MAP‐PET in bulk soil, LPOM, and MAOM across the top A and B horizons.
**Figure S14:** Structural equation modeling path diagrams for controls on mineral‐associated organic S (MAOS) concentrations, particulate organic S (POS) concentrations, proportion of MAOS in total organic S (f_MAOS_) in the A and B horizons (a‐c), average S oxidation states (ASOX) in bulk soil, LPOM and MAOM in the top A horizons (d–f), and ASOX in bulk soil and MAOM in the top B horizons (g, h). Numbers adjacent to arrows are standardized path coefficients, analogous to standardized regression coefficients. Red and blue arrows indicate positive and negative relationships, respectively. Arrow width is proportional to the strength of the relationship. The proportion of variance explained (*R* ^2^) is shown next to each response variable. Goodness of fit statistics are shown below each model, including Chi‐square/degree of freedom (χ^2^/df) test, the comparative fit index (CFI), and the root mean square error of approximation (RMSEA). *p < 0.05, **p < 0.01, ***p < 0.001. Predictor variables include mean annual temperature (MAT), effective soil moisture (mean annual precipitation minus potential evapotranspiration, i.e., MAP‐PET), mineral‐associated organic C (MAOC) concentration, particulate organic C (POC) concentration, proportion of MAOC in total organic C (f_MAOC_), ammonium oxalate‐extractable Fe and Al (FeAl_ox_) concentration, silt and clay (silt+clay) content, pH, C/N ratio of bulk soil (C/N_bulk_), C/N ratio of light particulate organic matter (C/N_LPOM_), C/N ratio of mineral‐associated organic matter (C/N_MAOM_).
**Figure S15:** Relationships between ester sulfate content in the MAOM fraction and (a) exchangeable Ca^2+^, (b) exchangeable Mg^2+^, and (c) soil cation exchange capacity (CEC). Exchangeable Ca^2+^ and Mg^2+^, as well as CEC, were determined using the ammonium acetate method (data provided by NEON).
**Figure S16:** The correlations of average S oxidation states (ASOX) in bulk soil, light particulate organic matter (LPOM) and mineral‐associated organic matter (MAOM) in the top A and B horizons with mean annual temperature (MAT) and effective soil moisture (mean annual precipitation minus potential evapotranspiration, i.e., MAP‐PET). Points are grouped by (A) soil order and (B) ecosystem type.
**Table S1:** Detailed soil profile descriptions for the NEON sites.
**Table S2:** Sulfur K‐edge XANES peak assignments, electronic oxidation states, and correction factors.
**Table S3:** The relative proportions of S species obtained from the Gaussian curve fitting results.

## Data Availability

The data that support the findings of this study are openly available in Figshare at https://doi.org/10.6084/m9.figshare.33931489. The deposited dataset includes site and soil‐profile information, all measured C, N, and S concentrations in bulk soil and the POM and MAOM fractions, exchangeable S concentrations (Table S1), the normalized S K‐edge XANES spectra of bulk soil, LPOM, and MAOM, and Gaussian curve fitting results (Table S3). Soil physical and chemical properties of the NEON Megapit horizons (pH, particle‐size distribution, bulk density, exchangeable cations, cation exchange capacity, and gypsum) were obtained from the NEON data product (DP1.00096.001; National Ecological Observatory Network 2026). Climate and productivity covariates (MAT, MAP, PET, and NPP) were obtained from WorldClim v2 (Fick and Hijmans 2017), Global‐AI_PET v3 (Zomer et al. 2022), and MOD17A3HGF v061 (Running and Zhao 2021).
