# Supplementary material for: Mineral Stabilization of Soil Organic Sulfur at the Continental Scale
Source: Glob Chang Biol. 2026 Oct 4;32(10):e71131. doi: 10.1111/gcb.71131 (PMC13635771; doi:10.1111/gcb.71131)
Supplement: Supplementary file 2 [file GCB-32-e71131-s002.zip › NEON S SI 09222026.docx]

Supporting Information for

**Mineral stabilization of soil organic sulfur at the continental scale**

Zhuojun Zhang^1,2^, M. Francesca Cotrufo^3^, Benjamin L. Turner^4^, Hai-Ruo Mao^2,5^, Chao Liang^6,7^, Mohsen Shakouri^8^, and Mengqiang Zhu^2,9^*

^1^ State Key Laboratory of Lake and Watershed Science for Water Security, Nanjing Institute of Geography and Limnology, Chinese Academy of Sciences, Nanjing, Jiangsu 211135, China

^2^ Department of Ecosystem Science and Management, University of Wyoming, Laramie, Wyoming 82071, United States

^3^ Department of Soil and Crop Science, Colorado State University, Fort Collins, Colorado 80523, United States

^4^ College of Resources and Environment, Shandong Agricultural University, Tai’an, Shandong 271000, China

^5^ Institute of Mountain Hazards and Environment, Chinese Academy of Sciences, Chengdu, Sichuan 610213, China

^6^ Zhejiang Key Laboratory of Soil Remediation and Quality Improvement, College of Environmental and Resource Sciences, Zhejiang A&F University, Hangzhou, Zhejiang 311300, China

^7^ Institute of Applied Ecology, Chinese Academy of Sciences, Shenyang, Liaoning 110016, China

^8^ Canadian Light Source Inc., University of Saskatchewan, Saskatoon, Saskatchewan S7N 2V3, Canada

^9^ Department of Geological, Environmental, and Planetary Sciences, University of Maryland, College Park, Maryland 20740, United States

* Corresponding author: Mengqiang Zhu

Email: mqzhu@umd.edu

**This PDF file includes:**

Figures S1 to S16

Tables S2 and S3

**Other supporting materials for this manuscript include the following:**

Table S1 (Excel file)

Sulfur K-edge XANES spectra data and Gaussian curve-fitting results (deposited in figshare: https://doi.org/10.6084/m9.figshare.33931489)

**Fig. S1.** Spatial distribution and Whittaker biome classification of the study sites. (a) Geographic distribution of sites, with symbols indicating soil order and colors indicating vegetation type. Map lines delineate study areas and do not necessarily depict accepted national boundaries. (b) Whittaker biome distributions of all sites (Whittaker, 1970). (c) Number of sites represented by each soil order and ecosystem type.

**Fig. S2.** Correlation between LPOS and POS in the topsoil.

**Fig. S3.** A comparative analysis of key organic S and soil characteristics across different ecosystems. (a-d) Total organic S (TOS), particulate organic S (POS), mineral-associated organic S (MAOS), and the proportion of MAOS in TOS (f_MAOS_); (e, f) exchangeable S concentration and its proportion of total S; (g-i) C/S ratios of bulk soil, particulate organic matter (POM), and mineral-associated organic matter (MAOM); (j-l) C-bonded S, ester sulfate, and average S oxidation state (ASOX) in bulk soil; (m–o) C-bonded S, ester sulfate, and ASOX in light particulate organic matter (LPOM); (p–r) C-bonded S, ester sulfate, and ASOX in MAOM; and (s–x) ammonium oxalate-extractable Fe and Al, dithionite-extractable Fe and Al, pH, soil organic C (SOC), C/N ratio, and silt + clay content, respectively. Different lowercase letters indicate significant differences among ecosystem types (one-way ANOVA followed by Tukey’s HSD, P < 0.05).

**Fig. S4.** Correlations between TOC (g/kg soil) and concentrations of MAOC and POC (g/kg soil) in the A and B horizons.

**Fig. S5.** Correlations between C/S and C/N ratios, as well as N/S and C/N ratios, in bulk soil, POM, and MAOM fractions.

**Fig. S6.** Correlation between f_MAOC_ and C/S_MAOM_.

**Fig. S7.** Relationships of MAOS concentration with (a) silt and clay (silt+clay) content and (b) ammonium oxalate-extractable Fe and Al (FeAl_ox_) concentration.

**Fig. S8.** Relationships between organic S pools and horizon thickness/depth. For A, B, and A+B horizons, MAOS, POS, and TOS stocks were calculated as organic S concentration × bulk density × horizon thickness.

**Fig. S9.** (a) Correlation between NPP and MAT, and (b) correlation between soil pH and MAP-PET.

**Fig. S10.** The chemical composition of organic S groups in bulk soil, LPOM and MAOM in the top A and B horizons.

**Fig. S11.** Correlations of the proportions of C-bonded S and ester sulfate with MAT and MAP-PET in bulk soil, LPOM, and MAOM across the top A and B horizons.

**Fig. S12.** Correlations of the proportions of the five Gaussian components (G1 - G5) with MAT in bulk soil, LPOM, and MAOM across the top A and B horizons. G1 represents reduced organic S, G2 thiophene-like S, G3 sulfoxide, G4 sulfonate, and G5 ester sulfate.

**Fig. S13.** Correlations of the proportions of the five Gaussian components (G1 - G5) with MAP-PET in bulk soil, LPOM, and MAOM across the top A and B horizons.

**Fig. S14.** Structural equation modeling path diagrams for controls on mineral-associated organic S (MAOS) concentrations, particulate organic S (POS) concentrations, proportion of MAOS in total organic S (f_MAOS_) in the A and B horizons (a-c), average S oxidation states (ASOX) in bulk soil, LPOM and MAOM in the top A horizons (d-f), and ASOX in bulk soil and MAOM in the top B horizons (g, h). Numbers adjacent to arrows are standardized path coefficients, analogous to standardized regression coefficients. Red and blue arrows indicate positive and negative relationships, respectively. Arrow width is proportional to the strength of the relationship. The proportion of variance explained (R^2^) is shown next to each response variable. Goodness-of-fit statistics are shown below each model, including Chi-square/degree of freedom (χ^2^/df) test, the comparative fit index (CFI), and the root mean square error of approximation (RMSEA). *p < 0.05, **p < 0.01, ***p < 0.001. Predictor variables include mean annual temperature (MAT), effective soil moisture (mean annual precipitation minus potential evapotranspiration, i.e., MAP-PET), mineral-associated organic C (MAOC) concentration, particulate organic C (POC) concentration, proportion of MAOC in total organic C (f_MAOC_), ammonium oxalate-extractable Fe and Al (FeAl_ox_) concentration, silt and clay (silt+clay) content, pH, C/N ratio of bulk soil (C/N_bulk_), C/N ratio of light particulate organic matter (C/N_LPOM_), C/N ratio of mineral-associated organic matter (C/N_MAOM_).

**Fig. S15.** Relationships between ester sulfate content in the MAOM fraction and (a) exchangeable Ca^2+^, (b) exchangeable Mg^2+^, and (c) soil cation exchange capacity (CEC). Exchangeable Ca^2+^ and Mg^2+^, as well as CEC, were determined using the ammonium acetate method (data provided by NEON).

**Fig. S16.** The correlations of average S oxidation states (ASOX) in bulk soil, light particulate organic matter (LPOM) and mineral-associated organic matter (MAOM) in the top A and B horizons with mean annual temperature (MAT) and effective soil moisture (mean annual precipitation minus potential evapotranspiration, i.e., MAP-PET). Points are grouped by (A) soil order and (B) ecosystem type.

**Table S2** Sulfur K-edge XANES peak assignments, electronic oxidation states, and correction factors.

| Peak | White-line energy (eV) | Electronic oxidation state (EOS) | Scaling factor | Spectral class | Representative compounds |
| --- | --- | --- | --- | --- | --- |
| G1 | 2472.05 ± 0.26 | -0.52 ± 0.16 | 0.76 ± 0.10 | Reduced organic S | thiols, sulfides, disulfides |
| G2 | 2473.28 ± 0.25 | 0.24 ± 0.15 | 1.21 ± 0.09 | Heterocyclic S | thiophenes |
| G3 | 2475.35 ± 0.16 | 1.52 ± 0.10 | 1.97 ± 0.06 | Sulfoxide-like S | sulfoxides |
| G4 | 2480.25 ± 0.18 | 4.55 ± 0.11 | 3.78 ± 0.07 | Sulfonate-like oxidized S | sulfonates |
| G5 | 2482.00 ± 0.12 | 5.62 ± 0.08 | 4.42 ± 0.05 | Sulfate-like S | ester sulfate, inorganic sulfate |

**Table S3** The relative proportions of S species obtained from the Gaussian curve fitting results.

| Sample name | G1 | G2 | G3 | G4 | G5 | Average oxidation state |
| --- | --- | --- | --- | --- | --- | --- |
|  | (%) | (%) | (%) | (%) | (%) |  |
| ABBY_A_bulk | 23.0 | 23.6 | 8.2 | 21.9 | 23.3 | 2.5 |
| BARR_Cgjj/Oajj_bulk | 45.4 | 26.8 | 8.5 | 10.3 | 9.0 | 0.9 |
| BART_A_bulk | 15.1 | 40.1 | 15.6 | 16.6 | 12.7 | 1.9 |
| BLAN_Ap1_bulk | 19.9 | 23.1 | 6.0 | 24.9 | 26.2 | 2.8 |
| CLBJ_A_bulk | 42.3 | 19.6 | 6.0 | 14.9 | 17.3 | 1.6 |
| CPER_A_bulk | 21.4 | 26.0 | 7.4 | 17.4 | 27.8 | 2.6 |
| DCFS_A1_bulk | 21.8 | 22.1 | 7.8 | 24.2 | 24.0 | 2.7 |
| DEJU_A_bulk | 31.0 | 20.3 | 9.2 | 14.3 | 25.2 | 2.2 |
| DELA_A1_bulk | 22.5 | 20.4 | 6.2 | 21.0 | 29.9 | 2.8 |
| GRSM_A1_bulk | 23.8 | 20.6 | 8.0 | 23.9 | 23.7 | 2.6 |
| GUAN_A_bulk | 19.3 | 18.1 | 6.0 | 24.7 | 32.0 | 3.2 |
| HARV_A_bulk | 15.9 | 22.5 | 6.9 | 26.5 | 28.2 | 3.1 |
| HEAL_A/Cjj_bulk | 41.9 | 26.2 | 9.8 | 12.8 | 9.4 | 1.1 |
| JERC_A_bulk | 26.9 | 34.4 | 21.9 | 5.5 | 11.3 | 1.3 |
| JORN_A_bulk | 29.6 | 32.1 | 15.7 | 8.0 | 14.6 | 1.5 |
| OAES_A_bulk | 15.6 | 12.1 | 3.1 | 20.6 | 48.5 | 3.9 |
| KONA_Ap_bulk | 26.7 | 19.9 | 5.0 | 22.1 | 26.3 | 2.6 |
| KONZ_A_bulk | 21.9 | 20.6 | 6.0 | 24.9 | 26.6 | 2.8 |
| LAJA_Ap_bulk | 24.9 | 15.3 | 4.2 | 20.4 | 35.2 | 3.0 |
| LENO_A_bulk | 27.2 | 24.1 | 9.2 | 19.4 | 20.1 | 2.2 |
| Mameyes_A_bulk | 12.9 | 14.0 | 4.3 | 14.8 | 54.0 | 4.0 |
| MLBS_A_bulk | 23.8 | 25.0 | 6.3 | 22.5 | 22.5 | 2.5 |
| MOAB_A_bulk | 25.4 | 18.6 | 6.5 | 19.9 | 29.7 | 2.7 |
| NIWO_A1_bulk | 17.6 | 18.7 | 8.3 | 27.5 | 27.8 | 3.1 |
| NOGP_A1_bulk | 20.8 | 22.5 | 7.8 | 21.6 | 27.3 | 2.8 |
| ONAQ_A1_bulk | 25.3 | 19.9 | 7.4 | 17.3 | 30.0 | 2.7 |
| ORNL_A_bulk | 18.8 | 23.8 | 7.2 | 23.8 | 26.4 | 2.8 |
| RMNP_A_bulk | 26.2 | 26.8 | 11.8 | 18.3 | 16.8 | 2.0 |
| SCBI_A_bulk | 22.4 | 23.8 | 7.9 | 23.5 | 22.4 | 2.6 |
| SERC_A_bulk | 24.9 | 24.1 | 7.9 | 20.3 | 22.7 | 2.4 |
| SJER_A1_bulk | 34.3 | 20.5 | 9.7 | 16.6 | 18.9 | 1.9 |
| SOAP_A1_bulk | 22.3 | 29.4 | 11.9 | 18.6 | 17.8 | 2.2 |
| SRER_A_bulk | 16.7 | 12.0 | 3.0 | 21.0 | 47.2 | 3.8 |
| STEI_A_bulk | 25.5 | 22.8 | 8.9 | 22.0 | 20.8 | 2.4 |
| STER_Ap1_bulk | 25.0 | 18.2 | 6.0 | 19.9 | 30.9 | 2.8 |
| TEAK_A_bulk | 25.6 | 28.6 | 11.1 | 17.6 | 17.1 | 2.0 |
| TOOL_Bg_bulk | 40.0 | 25.1 | 9.2 | 12.9 | 12.9 | 1.3 |
| TREE_A/E_bulk | 20.7 | 22.0 | 7.3 | 22.2 | 27.8 | 2.8 |
| UKFS_A1_bulk | 22.5 | 20.3 | 6.1 | 25.7 | 25.4 | 2.8 |
| UNDE_A_bulk | 22.9 | 25.7 | 11.0 | 21.2 | 19.2 | 2.3 |
| WOOD_AO_bulk | 25.9 | 23.1 | 8.2 | 21.1 | 21.6 | 2.4 |
| WREF_Ac_bulk | 15.0 | 10.0 | 3.1 | 12.5 | 59.4 | 4.2 |
| YELL_A_bulk | 27.0 | 24.9 | 10.2 | 19.3 | 18.7 | 2.1 |
| ABBY_Bw1_bulk | 37.2 | 6.5 | 1.4 | 9.4 | 45.5 | 2.9 |
| BARR_CgjjF2_bulk | 39.2 | 28.4 | 8.3 | 13.8 | 10.3 | 1.2 |
| BART_Bs1_bulk | 18.7 | 13.6 | 6.7 | 10.4 | 50.7 | 3.6 |
| BLAN_Bt1_bulk | 32.2 | 7.4 | 2.1 | 11.1 | 47.2 | 3.1 |
| BONA_Bg/Oajj_bulk | 37.4 | 20.6 | 7.3 | 19.2 | 15.6 | 1.8 |
| CLBJ_Bt1_bulk | 32.2 | 15.2 | 2.2 | 19.1 | 31.3 | 2.6 |
| CPER_Bt1_bulk | 27.4 | 11.9 | 2.3 | 17.5 | 40.8 | 3.2 |
| DCFS_Bw_bulk | 22.5 | 15.3 | 3.6 | 26.6 | 32.1 | 3.2 |
| DEJU_Bw_bulk | 23.1 | 2.8 | 4.2 | 13.2 | 56.7 | 3.9 |
| DELA_Bt_bulk | 19.8 | 11.1 | 2.4 | 11.4 | 55.3 | 3.8 |
| GRSM_Bw_bulk | 8.2 | 2.0 | 0.0 | 12.2 | 77.6 | 5.2 |
| GUAN_Bk1_bulk | 23.5 | 18.0 | 3.2 | 16.6 | 38.8 | 3.1 |
| HARV_Bw1_bulk | 8.5 | 5.4 | 1.5 | 24.6 | 60.1 | 4.8 |
| JERC_Bt1_bulk | 24.6 | 5.5 | 0.0 | 11.1 | 58.8 | 3.9 |
| JORN_Bw_bulk | 22.2 | 23.2 | 8.2 | 22.6 | 23.8 | 2.6 |
| OAES_Bw_bulk | 15.4 | 10.0 | 2.6 | 16.7 | 55.2 | 4.1 |
| KONA_Bt1_bulk | 34.9 | 19.6 | 4.1 | 18.6 | 22.9 | 2.1 |
| KONZ_Bt1_bulk | 25.6 | 16.2 | 4.0 | 25.2 | 29.0 | 2.9 |
| LAJA_Bssyz1_bulk | 1.3 | 0.8 | 0.0 | 6.1 | 91.8 | 5.8 |
| LENO_Bw_bulk | 25.6 | 18.6 | 5.8 | 22.2 | 27.9 | 2.7 |
| Mameyes_Bkk1_bulk | 7.1 | 3.7 | 0.0 | 7.9 | 81.3 | 5.2 |
| MLBS_Bg1_bulk | 42.5 | 10.2 | 2.0 | 14.4 | 30.9 | 2.2 |
| MOAB_Bw_bulk | 31.8 | 19.2 | 5.0 | 18.0 | 26.0 | 2.3 |
| NIWO_Bw1_bulk | 11.6 | 10.7 | 3.7 | 32.2 | 41.8 | 4.1 |
| NOGP_Bt1_bulk | 30.2 | 18.0 | 7.9 | 15.3 | 28.6 | 2.4 |
| ONAQ_Bw_bulk | 23.7 | 7.8 | 1.0 | 14.7 | 52.8 | 3.7 |
| ORNL_Bt1_bulk | 23.4 | 7.5 | 0.5 | 23.8 | 44.9 | 3.7 |
| PUUM_2Bw2_bulk | 14.0 | 13.9 | 5.2 | 29.4 | 37.6 | 3.8 |
| SCBI_Bt1_bulk | 21.4 | 4.5 | 0.2 | 9.5 | 64.5 | 4.2 |
| SERC_Bt1_bulk | 45.0 | 24.5 | 7.5 | 6.1 | 16.9 | 1.1 |
| SJER_Bw_bulk | 26.6 | 10.0 | 1.4 | 17.0 | 45.0 | 3.4 |
| SOAP_Bt1_bulk | 21.6 | 20.8 | 6.1 | 21.9 | 29.5 | 2.9 |
| SRER_Bw_bulk | 10.8 | 7.3 | 0.9 | 22.7 | 58.3 | 4.6 |
| STEI_Bs1_bulk | 17.1 | 16.8 | 6.0 | 22.4 | 37.7 | 3.4 |
| STER_Bt_bulk | 34.8 | 18.8 | 5.0 | 14.5 | 27.0 | 2.2 |
| TOOL_Cg_bulk | 33.5 | 25.8 | 7.2 | 13.8 | 19.7 | 1.8 |
| TREE_Bs_bulk | 18.5 | 17.3 | 7.0 | 24.7 | 32.5 | 3.2 |
| UKFS_Bt1_bulk | 25.8 | 12.9 | 2.3 | 28.1 | 30.9 | 3.1 |
| UNDE_BS1_bulk | 26.5 | 16.1 | 4.0 | 28.3 | 25.1 | 2.8 |
| WOOD_2Bw_bulk | 14.5 | 10.9 | 2.4 | 32.0 | 40.3 | 4.0 |
| WREF_Bw_bulk | 13.2 | 3.2 | 0.2 | 4.7 | 78.6 | 4.8 |
| YELL_Bt1_bulk | 24.4 | 20.0 | 6.6 | 23.4 | 25.6 | 2.7 |
| ABBY_A_MAOM | 27.7 | 26.4 | 9.1 | 18.7 | 18.1 | 2.1 |
| BARR_Cgjj/Oajj_MAOM | 43.6 | 28.8 | 8.4 | 11.8 | 7.4 | 0.9 |
| BART_A_MAOM | 19.5 | 42.0 | 13.9 | 14.7 | 9.9 | 1.6 |
| BLAN_Ap1_MAOM | 25.0 | 23.1 | 9.4 | 21.0 | 21.6 | 2.4 |
| CLBJ_A_MAOM | 38.8 | 17.8 | 7.5 | 12.2 | 23.7 | 1.9 |
| CPER_A_MAOM | 17.0 | 24.0 | 6.1 | 17.4 | 35.6 | 3.1 |
| DCFS_A1_MAOM | 23.0 | 18.9 | 8.3 | 25.1 | 24.6 | 2.8 |
| DEJU_A_MAOM | 21.9 | 17.1 | 9.0 | 17.6 | 34.4 | 3.0 |
| DELA_A1_MAOM | 33.0 | 23.5 | 2.7 | 13.9 | 26.9 | 2.2 |
| GRSM_A1_MAOM | 24.1 | 23.6 | 9.3 | 21.8 | 21.2 | 2.4 |
| GUAN_A_MAOM | 18.5 | 16.6 | 8.0 | 18.1 | 38.9 | 3.3 |
| HARV_A_MAOM | 22.0 | 27.4 | 9.2 | 21.3 | 20.2 | 2.4 |
| HEAL_A/Cjj_MAOM | 36.4 | 27.8 | 10.5 | 14.7 | 10.6 | 1.4 |
| JERC_A_MAOM | 32.0 | 28.0 | 21.9 | 5.8 | 12.3 | 1.3 |
| JORN_A_MAOM | 17.5 | 11.3 | 4.1 | 24.4 | 42.7 | 3.7 |
| OAES_A_MAOM | 23.3 | 8.6 | 2.7 | 15.7 | 49.7 | 3.6 |
| KONA_Ap_MAOM | 30.2 | 19.5 | 5.2 | 14.6 | 30.6 | 2.5 |
| KONZ_A_MAOM | 25.1 | 19.1 | 7.8 | 23.1 | 25.0 | 2.7 |
| LAJA_Ap_MAOM | 15.7 | 11.9 | 2.8 | 19.8 | 49.8 | 3.9 |
| LENO_A_MAOM | 35.6 | 27.0 | 14.3 | 11.9 | 11.2 | 1.3 |
| Mameyes_A_MAOM | 6.0 | 6.6 | 1.6 | 15.9 | 69.9 | 5.0 |
| MLBS_A_MAOM | 32.9 | 22.8 | 8.6 | 18.3 | 17.3 | 1.9 |
| MOAB_A_MAOM | 15.1 | 10.1 | 3.4 | 25.7 | 45.8 | 4.0 |
| NIWO_A1_MAOM | 21.5 | 17.4 | 9.1 | 26.8 | 25.2 | 2.9 |
| NOGP_A1_MAOM | 21.8 | 17.8 | 6.3 | 21.4 | 32.6 | 3.0 |
| ONAQ_A1_MAOM | 14.7 | 13.5 | 5.1 | 24.5 | 42.2 | 3.8 |
| ORNL_A_MAOM | 29.1 | 25.4 | 9.2 | 17.7 | 18.5 | 2.0 |
| RMNP_A_MAOM | 25.4 | 23.3 | 10.9 | 21.2 | 19.2 | 2.3 |
| SCBI_A_MAOM | 24.8 | 22.1 | 9.5 | 22.8 | 20.9 | 2.4 |
| SERC_A_MAOM | 31.4 | 24.5 | 8.7 | 16.6 | 18.9 | 1.9 |
| SJER_A1_MAOM | 16.9 | 16.8 | 10.8 | 26.0 | 29.5 | 3.2 |
| SOAP_A1_MAOM | 21.0 | 26.4 | 12.4 | 17.9 | 22.3 | 2.4 |
| SRER_A_MAOM | 24.8 | 10.0 | 2.3 | 23.3 | 39.6 | 3.4 |
| STEI_A_MAOM | 28.6 | 20.0 | 12.2 | 19.1 | 20.2 | 2.2 |
| STER_Ap1_MAOM | 15.1 | 11.8 | 5.4 | 20.5 | 47.1 | 3.9 |
| TEAK_A_MAOM | 38.1 | 19.8 | 8.8 | 15.3 | 17.8 | 1.7 |
| TOOL_Bg_MAOM | 29.4 | 24.5 | 10.1 | 17.5 | 18.4 | 2.0 |
| TREE_A/E_MAOM | 21.4 | 20.5 | 10.8 | 22.1 | 25.2 | 2.7 |
| UKFS_A1_MAOM | 27.9 | 20.6 | 6.3 | 23.2 | 21.9 | 2.4 |
| UNDE_A_MAOM | 30.7 | 36.9 | 20.0 | 6.9 | 5.6 | 1.0 |
| WOOD_AO_MAOM | 23.8 | 19.8 | 6.3 | 24.3 | 25.8 | 2.7 |
| WREF_Ac_MAOM | 23.3 | 12.5 | 4.8 | 10.8 | 48.7 | 3.4 |
| YELL_A_MAOM | 24.3 | 20.9 | 7.2 | 22.7 | 24.9 | 2.6 |
| ABBY_Bw1_MAOM | 36.5 | 11.0 | 3.3 | 12.0 | 37.2 | 2.6 |
| BARR_CgjjF2_MAOM | 30.4 | 25.0 | 7.3 | 20.8 | 16.5 | 2.0 |
| BART_Bs1_MAOM | 12.8 | 11.3 | 5.0 | 18.8 | 52.0 | 4.1 |
| BLAN_Bt1_MAOM | 20.4 | 11.3 | 2.4 | 14.4 | 51.4 | 3.7 |
| BONA_Bg/Oajj_MAOM | 37.5 | 20.8 | 6.2 | 19.3 | 16.1 | 1.8 |
| CLBJ_Bt1_MAOM | 21.8 | 12.5 | 5.1 | 8.7 | 51.8 | 3.5 |
| CPER_Bt1_MAOM | 27.6 | 12.0 | 3.4 | 12.7 | 44.3 | 3.1 |
| DCFS_Bw_MAOM | 26.8 | 17.3 | 4.5 | 20.8 | 30.7 | 2.8 |
| DEJU_Bw_MAOM | 12.1 | 13.8 | 5.0 | 17.9 | 51.2 | 4.0 |
| DELA_Bt_MAOM | 13.9 | 12.2 | 2.3 | 15.5 | 56.2 | 4.1 |
| GRSM_Bw_MAOM | 7.5 | 13.5 | 3.0 | 14.7 | 61.3 | 4.5 |
| GUAN_Bk1_MAOM | 15.8 | 10.3 | 2.4 | 21.3 | 50.1 | 4.0 |
| HARV_Bw1_MAOM | 13.5 | 6.7 | 2.6 | 24.5 | 52.6 | 4.3 |
| JERC_Bt1_MAOM | 14.0 | 0.0 | 0.0 | 22.3 | 63.7 | 4.8 |
| JORN_Bw_MAOM | 27.8 | 10.6 | 4.3 | 19.6 | 37.7 | 3.1 |
| OAES_Bw_MAOM | 16.4 | 10.0 | 1.1 | 16.2 | 56.3 | 4.1 |
| KONA_Bt1_MAOM | 27.9 | 15.2 | 3.9 | 15.7 | 37.5 | 2.9 |
| KONZ_Bt1_MAOM | 33.3 | 17.4 | 5.7 | 17.4 | 26.2 | 2.3 |
| LAJA_Bssyz1_MAOM | 5.2 | 1.3 | 0.0 | 12.0 | 81.5 | 5.4 |
| LENO_Bw_MAOM | 25.2 | 18.6 | 6.5 | 19.6 | 30.2 | 2.8 |
| Mameyes_Bkk1_MAOM | 2.7 | 2.9 | 0.4 | 8.0 | 85.9 | 5.6 |
| MLBS_Bg1_MAOM | 54.6 | 14.6 | 2.4 | 9.1 | 19.4 | 1.2 |
| MOAB_Bw_MAOM | 17.1 | 8.0 | 1.8 | 29.6 | 43.5 | 4.0 |
| NIWO_Bw1_MAOM | 17.9 | 14.4 | 5.5 | 25.5 | 36.6 | 3.5 |
| NOGP_Bt1_MAOM | 28.3 | 14.4 | 3.6 | 13.1 | 40.5 | 2.9 |
| ONAQ_Bw_MAOM | 32.9 | 16.3 | 8.4 | 6.9 | 35.6 | 2.4 |
| ORNL_Bt1_MAOM | 31.8 | 15.4 | 2.2 | 7.6 | 43.1 | 2.8 |
| PUUM_2Bw2_MAOM | 13.2 | 13.3 | 5.4 | 29.2 | 38.9 | 3.8 |
| SCBI_Bt1_MAOM | 21.0 | 6.9 | 2.2 | 7.6 | 62.3 | 4.0 |
| SERC_Bt1_MAOM | 21.0 | 4.3 | 3.3 | 13.3 | 58.1 | 4.0 |
| SJER_Bw_MAOM | 20.7 | 9.6 | 8.3 | 13.5 | 47.9 | 3.6 |
| SOAP_Bt1_MAOM | 38.1 | 14.8 | 4.7 | 12.6 | 29.7 | 2.2 |
| SRER_Bw_MAOM | 4.8 | 7.4 | 0.9 | 23.1 | 63.7 | 5.0 |
| STEI_Bs1_MAOM | 27.5 | 18.4 | 7.7 | 20.5 | 25.9 | 2.5 |
| STER_Bt_MAOM | 36.3 | 14.1 | 4.2 | 10.7 | 34.7 | 2.4 |
| TOOL_Cg_MAOM | 27.0 | 25.6 | 8.2 | 16.0 | 23.2 | 2.2 |
| TREE_Bs_MAOM | 16.0 | 13.4 | 7.7 | 25.0 | 38.0 | 3.6 |
| UKFS_Bt1_MAOM | 23.5 | 13.3 | 3.3 | 26.2 | 33.7 | 3.2 |
| UNDE_BS1_MAOM | 35.7 | 20.0 | 5.0 | 20.6 | 18.6 | 2.0 |
| WOOD_2Bw_MAOM | 13.6 | 8.5 | 1.5 | 34.8 | 41.5 | 4.2 |
| WREF_Bw_MAOM | 21.0 | 6.6 | 1.6 | 6.7 | 64.1 | 4.0 |
| YELL_Bt1_MAOM | 17.2 | 14.6 | 3.8 | 30.0 | 34.4 | 3.5 |
| ABBY_A LPOM | 32.0 | 31.1 | 12.6 | 14.7 | 9.6 | 1.4 |
| BARR_Cgjj/Oajj_LPOM | 45.1 | 29.3 | 10.3 | 9.9 | 5.5 | 0.7 |
| BART_A_LPOM | 26.4 | 38.3 | 14.1 | 13.8 | 7.4 | 1.3 |
| BLAN_Ap1_LPOM | 36.4 | 30.8 | 17.4 | 9.3 | 6.2 | 1.0 |
| BONA_Bg/Oajj_LPOM | 38.5 | 23.1 | 9.0 | 17.8 | 11.6 | 1.5 |
| CLBJ_A_LPOM | 41.0 | 28.1 | 12.9 | 11.4 | 6.6 | 1.0 |
| CPER_A_LPOM | 33.4 | 29.2 | 13.2 | 15.3 | 8.9 | 1.4 |
| DCFS_A1_LPOM | 29.0 | 27.3 | 10.6 | 19.0 | 14.0 | 1.8 |
| DEJU_A_LPOM | 29.4 | 33.0 | 15.7 | 12.2 | 9.7 | 1.4 |
| DELA_A1_LPOM | 31.5 | 34.7 | 14.8 | 11.3 | 7.7 | 1.2 |
| GRSM_A1_LPOM | 31.0 | 29.1 | 10.8 | 17.0 | 12.1 | 1.6 |
| GUAN_A_LPOM | 21.2 | 20.8 | 8.3 | 28.0 | 21.6 | 2.8 |
| HARV_A_LPOM | 24.5 | 30.9 | 9.8 | 19.7 | 15.0 | 2.0 |
| HEAL_A/Cjj_LPOM | 42.8 | 27.5 | 11.1 | 11.2 | 7.4 | 0.9 |
| JERC_A_LPOM | 24.3 | 34.7 | 14.7 | 15.3 | 10.9 | 1.6 |
| JORN_A_LPOM | 35.6 | 26.6 | 15.4 | 13.2 | 9.1 | 1.3 |
| OAES_A_LPOM | 26.5 | 22.4 | 10.2 | 21.8 | 19.1 | 2.3 |
| KONA_Ap_LPOM | 37.4 | 28.8 | 13.8 | 12.5 | 7.5 | 1.1 |
| KONZ_A_LPOM | 29.4 | 30.8 | 12.2 | 16.0 | 11.5 | 1.6 |
| LAJA_Ap_LPOM | 33.4 | 24.5 | 9.6 | 18.5 | 14.0 | 1.7 |
| LENO_A_LPOM | 31.8 | 32.4 | 12.7 | 14.1 | 9.0 | 1.3 |
| Mameyes_A_LPOM | 29.7 | 28.2 | 11.4 | 18.4 | 12.3 | 1.7 |
| MLBS_A_LPOM | 28.2 | 31.9 | 8.4 | 19.0 | 12.5 | 1.7 |
| MOAB_A_LPOM | 34.9 | 28.3 | 13.6 | 13.9 | 9.3 | 1.3 |
| NIWO_A1_LPOM | 25.7 | 25.0 | 12.0 | 21.2 | 16.0 | 2.1 |
| NOGP_A1_LPOM | 27.4 | 30.8 | 11.6 | 15.1 | 15.1 | 1.8 |
| ONAQ_A1_LPOM | 31.9 | 25.6 | 13.7 | 18.4 | 10.4 | 1.6 |
| ORNL_A_LPOM | 29.1 | 31.2 | 13.0 | 16.0 | 10.7 | 1.6 |
| PUUM_Bw1_LPOM | 23.1 | 20.7 | 6.6 | 26.8 | 22.8 | 2.7 |
| RMNP_A_LPOM | 28.9 | 32.9 | 14.5 | 14.9 | 8.9 | 1.4 |
| SCBI_A_LPOM | 31.1 | 31.4 | 13.3 | 15.3 | 9.0 | 1.4 |
| SERC_A_LPOM | 27.7 | 30.9 | 11.9 | 17.4 | 12.1 | 1.7 |
| SJER_A1_LPOM | 35.8 | 31.1 | 15.8 | 11.6 | 5.7 | 1.0 |
| SOAP_A1_LPOM | 25.0 | 35.0 | 17.0 | 15.6 | 7.3 | 1.5 |
| STEI_A_LPOM | 29.5 | 32.3 | 16.6 | 12.9 | 8.6 | 1.4 |
| STER_Ap1_LPOM | 34.0 | 28.4 | 12.2 | 15.3 | 10.1 | 1.4 |
| TEAK_A_LPOM | 26.5 | 31.7 | 13.6 | 16.3 | 12.0 | 1.7 |
| TOOL_Bg_LPOM | 45.8 | 29.9 | 12.1 | 7.2 | 5.0 | 0.6 |
| TREE_A/E_LPOM | 30.8 | 31.1 | 11.4 | 15.6 | 11.1 | 1.5 |
| UKFS_A1_LPOM | 31.1 | 27.5 | 11.4 | 17.6 | 12.4 | 1.7 |
| UNDE_A_LPOM | 33.4 | 31.2 | 14.5 | 12.9 | 8.0 | 1.2 |
| WOOD_AO_LPOM | 29.3 | 30.0 | 11.3 | 17.4 | 12.0 | 1.7 |
| WREF_Ac_LPOM | 23.1 | 34.6 | 15.4 | 13.4 | 13.4 | 1.7 |
| YELL_A_LPOM | 31.0 | 29.7 | 12.6 | 15.8 | 10.8 | 1.5 |

**References**

Whittaker, R. 1970. *Communities and ecosystems*: Macmillan.
